# Glycans Controlling Virus Infections: Meeting Report on the 1st International Symposium on Glycovirology Schöntal, Germany, 02–04 May 2018

**DOI:** 10.3390/v10110636

**Published:** 2018-11-15

**Authors:** Thilo Stehle, Thomas Peters, Laura Hartmann, Mario Schelhaas

**Affiliations:** 1 Interfaculty Institute of Biochemistry, University of Tübingen, D-72706 Tübingen, Germany; thilo.stehle@uni-tuebingen.de; 2 Research Group “ViroCarb: Glycans controlling non-enveloped virus infections”, Coordinating University of Tübingen, D-72706 Tübingen, Germany; 3 Institute of Chemistry and Metabolomics, University of Lübeck, D-23562 Lübeck, Germany; thomas.peters@uni-luebeck.de; 4 Institute of Organic Chemistry and Macromolecular Chemistry, Heinrich-Heine-University Düsseldorf, D-40225 Düsseldorf, Germany; laura.hartmann@hhu.de; 5 Institute of Cellular Virology, ZMBE, University of Münster, D-48149 Münster, Germany

**Keywords:** glycan, virus, infection, structural biology, chemical biology, cell biology

## Abstract

Glycans are, with nucleic acids, proteins and lipids, one of the four founding structures of cellular life. Due to their non-template synthesis, they are inherently heterogeneous and difficult to study with regards to their structure and function. Since 2016, the research group ViroCarb, funded by the German Research Foundation, has investigated the role of glycans in non-enveloped virus infections with a highly interdisciplinary approach. The core idea was to bring together scientists and students from various disciplines such as structural biology, cell biology, virology and chemistry to advance research by an interdisciplinary means. In 2018, ViroCarb hosted the 1st International Symposium on Glycovirology in Schöntal, Germany, with a similar aim. Scientists from various disciplines gathered to discuss their area of study, present recent findings, establish or strengthen collaborations, and mentor the next generation of glycovirologists through formal presentations and informal discussions. The secluded meeting at the monastery of Schöntal gave ample time for in-depth discussions. On behalf of ViroCarb, this report summarizes the reports and highlights advances in the field.

## 1. Introduction

Glycans are carbohydrate structures that decorate all cell surfaces and most secreted proteins of vertebrates and higher invertebrates. They are attached to either proteins or lipids, and they act as ligands for many glycan-binding host proteins. Known as lectins, such proteins play crucial roles in the function of cells, organs, and the immune system of humans and other mammals. Glycans take part in diverse biological processes, including cell-cell recognition, cell growth and differentiation, neoplastic transformation and cell death. The precise function of glycans in many of these processes is poorly understood, in part due to the limited availability of biologically relevant synthetic glycans, and technical challenges in their analysis, including interactions with proteins. Many difficulties that are associated with the investigation of glycan-protein interactions are caused by the transient (low-affinity) range of many such interactions, the naturally occurring chemical diversity of glycans, and the fact that they are secondary gene products. Thus, they cannot be specifically targeted with many standard cell biology tools (e.g., knock-out technology and RNA silencing).

Viruses use highly sophisticated and complex strategies to mount infections and to modulate host responses. Specific knowledge on the interactions between viruses and protein receptors or attachment factors is available for many viruses (reviewed in [1,2,3,4]). In some cases, such knowledge has led to sophisticated models involving conformational changes in viral proteins and receptors as a result of receptor engagement (e.g., [5,6,7,8,9,10]. By contrast, the roles of protein-glycan interactions in viral attachment and entry are less well understood, in part because cell-surface glycans form a heterogeneous mixture of complex carbohydrate moieties that are difficult to classify. For many viruses, only fragments of glycan receptors such as terminal sialic acid (Sia), sialyllactose, or sulfated oligosaccharides have been identified, and it is entirely unknown to which cell-surface glycoconjugates these fragments belong. As a consequence, it is largely unclear how glycan-binding impacts post-attachment events in the life cycle of most viruses, such as cell entry and viral uncoating.

Although many viruses have been known for some time to use cell-surface carbohydrates to initiate infection, our understanding of these interactions remains fragmented. Only recently, advances in glycan microarray screening technology have rapidly accelerated the identification of specific glycan receptors [11]. It is now also possible to map glycan epitopes that bind to a virus in solution using saturation transfer difference (STD) NMR spectroscopy [12,13,14,15], define the atomic level structure of the virus-glycan interaction using X-ray crystallography, use virus-like particles (VLPs) or pseudoviruses (assembled virus particles that lack the correct genome and are therefore non-infectious) to analyze the determinants of recognition, and design mutations to determine the precise effect of glycan-binding in disease pathogenesis. Moreover, modern mass spectrometry (MS) techniques have advanced such that native MS can be used to study the dependence of glycan-binding on assembly state [16], and both epitopes and conformational changes for different assembly states can be mapped with hydrogen/deuterium exchange MS (HDX MS) [17].

Collectively, these advances enable a thorough structural and functional analysis of virus-glycan interactions that was simply not possible just a few years ago. The expanding knowledge on glycan variety and structures as well as the increasing availability of glycan probes advances the possibilities of infection studies. Development of easy, versatile methods to study virus entry in high-throughput mode, will eventually allow the screening of antiviral compounds in a fast and cost-effective manner.

**Figure 1:**
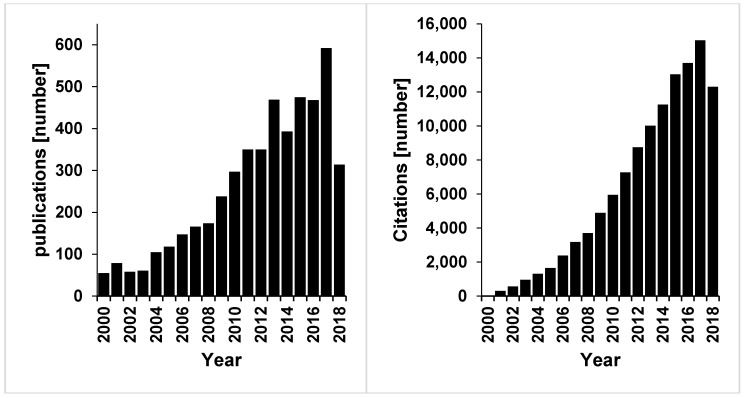
Web of Science search illustrating the increase in research activity in the field of glycovirolgy from 2000 to now (search terms: TOPIC: glycan AND TOPIC: virus). Left: Number of publications; right: Number of citations. Databases searched: WOS, BCI, CCC, DRCI, DIIDW, KJD, MEDLINE, RSCI, SCIELO, ZOOREC.

The research on glycan-virus interactions constitutes a steadily growing field of high importance. A search on the Web of Science displays the steady increase in publications and their citation over the last two decades (Figure 1). However, no obvious forum existed that would bring together researchers with the highly divergent expertise that is needed to tackle the questions arising in this field. Therefore, the research unit ViroCarb funded by the German Research Foundation (DFG), hosted the “1st International Symposium on Glycovirology” in Schöntal, Germany in 2018. The main aim of the symposium was to bring together structural biologists, chemists, physicists, biologists and virologists, but who would normally not meet each other because they usually attend conferences focused on a specific pathogen or methodology. As a result, this meeting was truly interdisciplinary. In the following, the authors of this report will showcase and highlight examples of the research discussed at the meeting, each from their unique viewpoint of expertise.

## 2. Thilo Stehle, University of Tübingen

As a structural biologist interested in studying glycan-driven mechanisms of viral attachment and entry, I was particularly fascinated by talks that addressed the identification of glycan receptors, and by studies that exemplified different strategies to modulate, and interfere with, virus-glycan interactions.

The presentations by **Rick Cummings** and **Ten Feizi** focused on the use of glycan microarrays to study binding profiles and specificities of glycan-binding viral proteins. These two talks nicely showcased the usefulness of glycan arrays to identify ligands, and to discern finer differences in virus–glycan binding preferences by enabling rapid, high-throughput screening of several glycans as potential virus ligands. Glycan array screening is analogous to the more familiar microarrays that are used to study gene expression. Glycans are immobilized on an array and then incubated with whole virus or the viral attachment protein to identify specific glycan receptors for viruses, and to compare glycan-binding preferences of different virus strains. Although arrays from the different platforms vary in the composition of the glycans on the array, as well as the glycan-coupling method, different types of arrays complement each other, and they have often enabled structural studies.

A particularly striking example for the application of this technology was presented by **Niklas Arnberg**, who showed in his talk that glycan array screening has been essential to identifying the glycan receptor of species D adenoviruses. It had been known for many years that some species D adenoviruses bind receptors terminating in sialic acid, but the nature of the glycan had remained elusive, until glycan array screening revealed that the fiber knob of the species D adenovirus Ad37 specifically recognizes the oligosaccharide GD1a, a disialylated compound with two branches that each terminate in sialic acid. Subsequent structural analyses of the trimeric Ad37 fiber knob in complex with GD1a, established that the two terminal sialic acid residues bind to two different Ad37 fiber knob protomers within a trimer in an identical manner, thus engaging two of three possible binding sites in the knob. With this information, the Arnberg group was able to design trivalent inhibitors that engage all three possible binding sites, and thus, bind to the fiber knob with high affinity. It is likely that this design of a multivalent ligand can also be adapted for the inhibition of other glycan-binding viruses, as viruses typically have multimeric proteins in their capsids.

In addition to being human pathogens, adenoviruses are also useful vectors for gene delivery, as they can package foreign DNA and deliver it to specific locations within the infected host. **Rita Gerardy-Schahn** presented a study in which her group replaced the entire fiber knob of an adenovirus with a protein that recognizes polysialic acid. This glycan is rare in adult humans, but it is highly expressed in malignant tumors such as glioma and small cell lung cancer. The modified viruses have polysialic acid-dependent infection modes and a strong oncolytic capacity with polysialic acid positive cells in culture. Therefore, they might be useful to specifically deliver oncolytic adenoviruses to tumor cells.

A different strategy for glycan-based inhibition of viruses was presented by **Mark von Itzstein**, who explored the potential of sialic acid variants that carry additional sulfonate groups as inhibitors for the influenza virus sialidase. His talk highlighted a new direction for influenza virus sialidase inhibitor development, as sulfonates can be synthesized efficiently and have potent inhibitory effects. This current work builds on earlier studies that lead to the development of the commercially-available neuraminidase inhibitor, oseltamivir.

The talks highlighted above were embedded among many other outstanding presentations on various aspects of glycobiology and virology. For me, one of the most important values of the conference was being educated about the range of state-of-the art approaches available to study virus-glycan relationships. Many conference participants felt similarly, and frequently commented on how much they had learned at this particular symposium and how they had been inspired by the talks.

## 3. Thomas Peters, University of Lübeck

Having studied carbohydrate-protein interactions with high-resolution NMR spectroscopy for many years, I was particularly enthusiastic about the interdisciplinary aspects that the International Glycovirology symposium offered. Many presentations highlighted how X-ray crystallography over the past decades has become a driving force for the identification and atomic-level characterization of virus-glycan recognition. In fact, aspects of structural biology were important for the majority of studies presented at this meeting. Presentations by **Mark von Itzstein**, **Bärbel Blaum**, and **Grant Hansman** nicely documented the power of this methodology. In particular, **Grant Hansman** presented high-resolution crystal structure data on a norovirus capsid protein complexed with specific nanobodies. Nanobodies can be produced in very good yields in a variety of expression systems, display great stability, and specifically recognize given epitopes with nanomolar or subnanomolar affinity, turning them into powerful potential therapeutics. Hansman’s work specifically addressed inhibition of HBGA-binding to noroviruses, possibly providing novel and promising antiviral strategies.

Another outstanding contribution was the presentation of **Tom Smith**. Using cryo electron microscopy (cryo EM), the group has shown that viral capsid proteins are much more flexible than suggested by static pictures from crystallography. In the example of norovirus-host interactions, the presence of bile acids leads to a significant conformational rearrangement of the capsid. In the future, it will be important to study underlying molecular mechanisms with complementary biophysical techniques capable of characterizing the different time scales of motions responsible for such rearrangements.

Several presentations addressed the development of cell culture systems to evaluate compounds e.g., novel antivirals. Significant progress has been made in this field, as was highlighted in talks by **Steeve Boulant** and **Julie Pfeiffer**. It appears that human intestinal organoids are a powerful tool to study gastrointestinal viruses, such as noroviruses.

The talks of **Ten Feizi**, **Rick Cummings** and **Geert-Jan Boons** made clear that the investigation of glycans involved in viral infections involves a unique challenge not present in other areas of virology. This challenge is the availability of pure and defined glycan chains for such things as the use in micro arrays. Glycans cannot easily be synthesized using recombinant techniques. **Geert-Jan Boons** showed how classical carbohydrate chemistry can be combined with recombinant glycosyltransferases to obtain defined glycan arrays. **Rick Cummings** demonstrated how such technology can be applied to identify high-affinity complex glycans binding to rotavirus capsid proteins.

From my point of view, the availability of defined and chemically modified glycans remains a substantial challenge in glycovirology research. Especially for the use with more demanding biophysical techniques, such as crystallography or NMR, this is an issue. Last but not least, this problem surfaces again, with the investigation of glycosphingolipids as presented by **Ludger Johannes**. Glycosphingolipids are notoriously difficult to obtain in pure form or large amounts. Upcoming glycovirology meetings will hopefully devise novel paths to solve the problem of glycan availability.

## 4. Laura Hartmann, University of Düsseldorf

While the identification of natural glycans and their role viral adhesion remains a challenging task, researchers have been able to create glycan-mimetic compounds and to derive glycan-based drugs such as antivirals through selective chemical variations. Both topics have been covered at the conference, with **Mark von Itzstein** opening the discussion with his lecture on the synthesis and evaluation of sulfonated sialic acid derivatives as potent inhibitors of influenza virus replication. Rather than modifying natural glycans, **Geert-Jan Boons** presented a cell-surface engineering approach introducing different oligosaccharides into cell surfaces that usually do not include such motifs, allowing for new insights into lassa virus infectivity. While it remains a challenging task to identify glycans involved in viral adhesion, we can already now start asking how many of these natural structures are required or could be replaced by other chemical entities, enabling high avidity. Members of the organizing research consortium also contributed to the discussion: **Grant Hansman** showed the binding of non-glycan motifs such as citric acid to norovirus capsids, and **Mario Schelhaas** introduced glycopolymers synthesized by the group of **Laura Hartmann**, as inhibitors of viral adhesion for papillomaviruses and a variety of other glycan-binding virus families.

Gaining new insights into the role of glycans in viral adhesion is also promoted by novel assays and analytical methods looking at specific features of glycan receptors, such as the importance of multivalency when engaging viral interactions. In her lecture, **Alison Ashcroft** showed the power of mass spectrometry when investigating the structure and assembly of viruses. **Marta Bally** presented a novel set-up using TIRF microscopy to investigate adhesion and movement of virus particles on GAG-functionalized surfaces depending on the type of GAG, and their degree of sulfation. Going from immobilized glycans to membrane-anchored systems, **Raluca Groza** from the **Helge Ewers** group, showed the latest data on the set-up of a free-standing bilayer and tracking of SV40 virus-like particles, to investigate effects of membrane deformation during viral adhesion. Looking at the dynamics of glycan-virus interactions from a kinetic point-of-view is possible using biolayer interferometric analysis, as presented by **Xander de Haan**, showing the latest results on influenza A virus binding and rolling controlled by a hemagglutinin/neuraminidase balance. While many of these assays use natural glycan structures, the investigation of glycan mimetics could help to better understand structure-property correlations and potentially derive novel strategies for antiviral treatment. The lively discussions after the talks showed that the meeting indeed facilitated communication between scientists from multiple areas and might have sparked new collaborations.

## 5. Mario Schelhaas, University of Münster

Being a chemist by training and having been reeducated as a cell biologically-interested virologist, I have been impressed by the interdisciplinary nature of this meeting. Despite my awareness of that fact, the most personal revelation for me was to experience the breadth of methodology developed, and successfully employed, in studying pathogen-glycan biology in one meeting. Like the handle of a corkscrew, recent methodology development provides the power to dig deep and “uncork” hitherto inaccessible functional aspects of glycan biology.

As has already been discussed above in talks by **Gert Jan Boons**, **Mark von Itzstein**, **Ten Feizi**, and **Laura Hartmann** from the ViroCarb research unit, the event highlighted chemical approaches to synthesize naturally occurring, as well as chemically-modified glycans, and incorporating them into biological systems. This allowed insights into the infection of viruses and provided rationales, leads or even fully developed substances to fight such infections. On top of its great medical importance, it also helps us to fundamentally understand the principles of virus-glycan engagements.

The continuing refinement of analysis approaches - from mass spectrometry over STD-NMR to innovative microscopy, combined with the use of integrated computational pipelines highlighted in talks and posters from **Alison Ashcroft**, **Ludger Johannes**, and the **Uetrecht** and **Peters** groups—likewise helps to unravel more details of biological processes.

Exemplary for the study of biological functions, I would like to showcase three presentations. **Julie Pfeiffer** gave a riveting talk on how gut bacteria enhance enteric virus infectivity. Her work pioneered and established, together with the work of others, a new paradigm by which enteric viruses transiently engage bacterial surface polysaccharides. This not only enhances delivery through the glycocalyx to target cells, but also enhances viral fitness through the stabilization of virions. **Raluca Groza** from the Ewers lab made use of a free-standing membrane system to address how polyomavirus engagement of glycosphingolipids impacts the dynamics of the virus in the plane of the membrane, and how it affects the deformation process that can eventually lead to formation of membrane tubules akin to endocytic structures. The formation of these structures had been initially described for bacterial toxins by **Ludger Johannes** and his team. He expanded on this topic and showed that not only can pathogens and viruses use this mechanism to generate their “own” endocytic pits, but that cellular lectins can also drive membrane curvature changes and the formation of endocytic pits. His results further implied that cortical actin polymerization and fluctuation forces contribute to the clustering of glycosphingolipid-lectin complexes, a prerequisite to curvature generation. As such, work on glycan-pathogen interactions allows the discovery of biophysical properties of glycosphingolipids important for cellular physiology.

## 6. Concluding Remarks

The showcases described above represent only a glimpse of the 21 talks and 29 posters presented at this meeting. All selected posters and talks were of high quality and novelty. But what made this meeting a most worthwhile experience were the lively discussions sparked by those contributions. Confined in the beautiful monastery of Schöntal, chemists, biologists, and virologists pooled ideas and developed concepts to be tested in the coming months and years. Those investigations will be fueled and driven by new collaborations, as this meeting has also served as a matchmaker of sorts. Beside the scientific presentations, a plenary discussion was held to review important directions in the field. This discussion yielded, as one of the most critical points, the need for continued interdisciplinary cooperation, and the urge to provide for future symposia of this kind as a venue for open discussion and collaboration.
